# Combined Serum IL-6 and CYFRA 21-1 as Potential Biomarkers for Radon-Associated Lung Cancer Risk: A Pilot Study

**DOI:** 10.3390/biomedicines13092145

**Published:** 2025-09-03

**Authors:** Narongchai Autsavapromporn, Aphidet Duangya, Pitchayaponne Klunklin, Imjai Chitapanarux, Chutima Kranrod, Churdsak Jaikang, Tawachai Monum, Shinji Tokonami

**Affiliations:** 1 Division of Radiation Oncology, Department of Radiology, Faculty of Medicine, Chiang Mai University, Chiang Mai 50200, Thailand; aphidet.d@cmu.ac.th (A.D.); pitchayaponne.kl@cmu.ac.th (P.K.); imjai.chitapanarux@cmu.ac.th (I.C.); 2 Institute of Radiation Emergency Medicine, Hirosaki University, Hirosaki, Aomori 036-8564, Japan; kranrodc@hirosaki-u.ac.jp (C.K.); tokonami@hirosaki-u.ac.jp (S.T.); 3 Toxicology Section, Department of Forensic Medicine, Faculty of Medicine, Chiang Mai University, Chiang Mai 50200, Thailand; churdsak.j@cmu.ac.th (C.J.); tawachai.m@cmu.ac.th (T.M.)

**Keywords:** biomarkers, lung cancer, non-smoking, radon, IL-6, CYFRA 21-1

## Abstract

**Background**: Radon, a naturally occurring radioactive gas, is increasingly recognized as a major risk factor for lung cancer (LC), especially among non-smokers. The objective of this study was to identify serum biomarkers for the early detection of LC in individuals at high risk due to prolonged residential radon exposure in Chiang Mai, Thailand, and to assess whether the use of single or combined biomarkers improves the sensitivity and specificity of detection. **Methods**: A total of 15 LC patients and 30 healthy controls (HC) were enrolled. The HC group was further stratified into two subgroups: low radon (LR, n = 15) and high radon (HR, n = 15) exposure. All participants were non-smokers or former smokers. Serum levels of cytokeratin 19 fragment (CYFRA 21-1), carcinoembryonic antigen (CEA), interleukin-6 (IL-6), interleukin-8 (IL-8), transforming growth factor-alpha (TGF-alpha), and indoleamine 2,3-dioxygenase-1 (IDO-1) were measured using the Milliplex^®^ Kit on a Luminex^®^ Multiplexing Instrument (MAGPIX^®^ System). **Results:** Serum CEA, IL-6 and IL-8 levels were significantly higher in LC patients compared to the HC group (*p* < 0.05). Among analyzed biomarkers, only IL-8 was significantly elevated in LC patients compared to the HR group (*p* = 0.04). Notably, CYFRA 21-1 was the only biomarker that significantly differed between LR and HR groups (*p* = 0.004). The diagnostic potential of these biomarkers was evaluated using receiver operating characteristic (ROC) analysis. Individually, IL-6 showed the highest discriminative ability for differentiating LC patients from both HC and HR groups, with high specificity but moderate sensitivity. Combining IL-6 and IL-8 improved specificity and increased the area under the ROC curve (AUC), though it did not enhance sensitivity for distinguishing LC from HC. For distinguishing LC from HR individuals, IL-6 and CYFRA 21-1 exhibited strong diagnostic performance. Their combination significantly improved diagnostic accuracy, yielding the highest AUC, sensitivity, and specificity. In contrast, CEA, IL-8, TGF-alpha, and IDO-1 demonstrated limited diagnostic utility. **Conclusions:** Based on the available literature, this is the first study to evaluate the combined use of IL-6 and CYFRA 21-1 as potential biomarkers for LC screening in individuals with high residential radon exposure. Our findings highlight their utility, particularly in combination, for improving diagnostic accuracy in this high-risk population.

## 1. Introduction

Lung cancer (LC) ranks as the leading cause of cancer mortality worldwide [1]. In Thailand, approximately 23,494 new LC cases and 19,864 deaths were reported in 2022 [2]. Non-small cell lung cancer (NSCLC) accounts for approximately 80–85% of all LC cases, with the remainder classified as small cell lung cancer (SCLC). NSCLC primarily consists of three histological subtypes: adenocarcinoma, squamous cell carcinoma, and large cell carcinoma [3]. Despite advances in LC treatments such as particle beam therapy, targeted therapy, immunotherapy, and hormone therapy, the prognosis for NSCLC remains poor, with a 5-year survival rate of only 28% [4]. Early screening and diagnosis are therefore essential to improve prognosis and therapeutic outcomes. Currently, low-dose computed tomography (LDCT) is the standard screening method; however, it is associated with radiation exposure, high cost, and a considerable rate of false positives. Serum biomarkers offer a non-invasive and cost-effective method for the early detection of NSCLC [5]. Several serum biomarkers, including Cytokeratin 19 Fragment (CYFRA 21-1), Carcinoembryonic Antigen (CEA), and Interleukin-6 (IL-6), have been investigated for their potential in diagnosing NSCLC [6,7,8]. However, their limited sensitivity and specificity limit their effectiveness as standalone biomarkers for screening purposes [9]. The absence of highly specific NSCLC biomarkers suggests that relying on a single serum marker may not achieve sufficient diagnostic accuracy and reliability.

Chiang Mai province serves as the capital of upper northern Thailand. According to a World Health Organization (WHO) report in 2020, LC ranked second in both incidence and mortality among men and women in this region [10]. The high incidence of LC in Chiang Mai province is primarily associated with cigarette smoking, exposure to secondhand smoke, environmental exposure to fine particulate matter (PM), and prolonged indoor radon inhalation [11]. Radon (^222^Rn) is a naturally occurring radioactive gas produced by the decay of uranium-238 (^238^U) found in soil, rock, groundwater, air (including PM), and building materials. It is a colorless, odorless, invisible gas with a half-life of 3.82 days [12]. In accordance with the International Agency for Research on Cancer (IARC), radon is classified as a Group 1 human carcinogen. It is the second leading cause of LC following cigarette smoking and the primary cause of LC among non-smokers [13].

Previous studies have shown that indoor radon activity concentrations in Chiang Mai province exceed the global average, with significantly elevated levels observed during periods of severe air pollution, particularly during high PM seasons [14]. Consequently, developing effective screening and diagnostic methods for individuals with long-term residence in areas of elevated radon concentration is crucial for identifying those at risk of developing LC. Such methods should ideally be non-invasive, highly accurate, reliable, and cost-effective. Our previous studies identified serum CYFRA 21-1 and CEA as promising biomarkers for LC in individuals chronically exposed to high radon levels [15]. However, further validation of these biomarkers is required. Additionally, exploring other serum biomarkers or their combinations may improve diagnostic accuracy. Therefore, this study aimed to identify serum biomarkers for early detection of LC in individuals at high risk due to prolonged residential radon exposure, and to evaluate whether single or combined biomarkers could enhance the sensitivity and specificity of LC diagnosis. We hypothesize that combining multiple serum biomarkers will achieve the sensitivity and specificity necessary to effectively predict LC risk in populations chronically exposed to high radon levels.

## 2. Materials and Methods

### 2.1. Study Area

Kong Khaek, a subdistrict in Mae Chaem district, is situated in the southern region of Chiang Mai province. This area experiences some of the highest PM levels in Chiang Mai and is distinguished by forested landscapes and granite highland mountains, which serve as a source of radon gas. It comprises 12 villages with a total population of 6572 residents and 2304 households as of 2023. To assess indoor radon levels, a passive radon-thoron discriminative monitor (RADUET, Radosys Ltd., Budapest, Hungary) was deployed from September 2022 to March 2023 [16]. The recorded indoor radon activity concentrations varied between 18.5 and 119 Bq/m^3^, with a mean value of 40.8 ± 22.6 Bq/m^3^. The average concentration of indoor radon in this region exceeds both national and global average [17]. Based on these findings, participants were classified into three groups according to the radon levels in their homes: low (<30 Bq/m^3^), moderate (30–50 Bq/m^3^), and high (>50 Bq/m^3^). All participants had resided in this area for at least 15 consecutive years, ensuring sufficient cumulative exposure to indoor radon to support the scientific validity of the study. It is well recognized that even low-dose radiation, when sustained over extended periods, can increase the risk of lung carcinogenesis [12].

### 2.2. Study Subjects and Sample Collection

The Human Research Ethics Committee of the Faculty of Medicine at Chiang Mai University, granted ethical approval for this study (Research ID: 8613) on 5 July 2022. A transitional study was conducted on a selection of Kong Khaek residents who had lived in the area for at least 15 years. A total of 135 individuals participated, including 50 LC patients and 85 healthy controls (HC). The HC group comprised 45 individuals from the low-radon (LR) exposure group and 40 from the high-radon (HR) exposure group. LC patients were enrolled from the Division of Radiation Oncology, Department of Radiology, Faculty of Medicine, Chiang Mai University, between September 2022 and August 2023 and resided in areas where indoor radon levels had been previously measured [14,15,17]. Only patients with NSCLC were included; there were no restrictions on histology subtype, cancer stage, or gender. The inclusion criteria for all study groups were: (I) age between 18 and 80 years, (II) no prior history of chemotherapy or radiotherapy, (III) non-smoking status or former smokers who had quit more than 20 years ago. The exclusion criteria for all groups included: (I) pregnancy, (II) chronic inflammatory diseases, and (III) a history of other cancers. Consequently, 15 participants from the LC group and 30 participants from the HC group (15 from the LR and 15 from the HR groups), matched by age and gender, were selected for further analysis (Table 1). The selection process for participants included in the serum study was informed by metabolomics data, as this approach enabled the identification of distinct metabolite differences among LC, LR, and HR groups [18,19].

All participants received an explanation of the study’s purpose and gave written informed consent before completing a questionnaire and donating blood samples. The questionnaire, specifically developed for this study by our research team, collected data on smoking history, alcohol intake, air pollution exposure, dietary patterns, family cancer history, and occupational background. Blood samples (10 mL) were obtained from all participants after 10–12 h fasting in the early morning by a trained nurse. Serum was extracted through centrifugation at 3000× *g* for 10 min (4 °C) and stored at −80 °C for further analysis. All samples were processed within two hours of collection. For LC patients, please note that blood samples were obtained prior to the initiation of radiation therapy to avoid any potential influence of treatment on biomarker levels.

### 2.3. Determination of Biomarker Serum Levels

Assays for CYFRA 21-1, CEA, IL-6, Interleukin-8 (IL-8), and Transforming Growth Factor Alpha (TGF-alpha) (Cat. No. HCCBP1MAG-58K), as well as Indoleamine 2,3-Dioxygenase 1 (IDO-1) (Cat. No. HCKP2-11K-01), were performed using the Milliplex^®^ Kit from Merck Millipore (St. Louis, MO, USA) on a Luminex^®^ Multiplexing Instrument (MAGPIX^®^ System), following the manufacturer’s instructions [20]. Assay plates were first washed with wash buffer, sealed, and then mixed on a plate shaker at room temperature for 10 min. After decanting the wash buffer, 50 μL of diluted standards, quality controls, and serum samples (25 μL) were dispensed into the designated wells. The plates were subsequently incubated overnight at 4 °C on a plate shaker in the presence of fluorescently labeled capture antibody-coated beads to detect CYFRA 21-1, CEA, IL-6, IL-8, TGF-alpha and IDO-1. After incubation, the contents of each well were removed and washed with the aid of a handheld magnet. Then, 50 μL of biotinylated detection antibodies were introduced into each well, followed by a 1-h incubation at room temperature with gentle shaking. Without discarding the contents, 50 μL of streptavidin–phycoerythrin was added. The mixture was incubated for 30 min at room temperature, washed again using the handheld magnet, and resuspended in sheath fluid. Finally, each serum sample was analyzed in triplicate using the Luminex xPONENT software, version 4.2 (Luminex Corp., Austin, TX, USA), and the results were expressed in picograms per milliliter (pg/mL).

### 2.4. Statistical Analyses

The measurement data were presented as the mean ± standard deviation. All measurements were above the limit of detection (LOD), and no missing data were observed, likely reflecting the high sensitivity of the assay and rigorous sample handling. The analysts were not blinded to group allocation, which is acknowledged as a limitation of this study. Since all samples were processed within a single batch, no batch-effect adjustment was required. Comparisons between two groups were analyzed using the Mann–Whitney U test, while the Chi-square test and Kruskal–Wallis test were applied for comparisons among three groups. Receiver operating characteristic (ROC) curve analysis was performed to evaluate the diagnostic value of biomarkers. The optimal cutoff value, along with the corresponding sensitivity (true positives/true positives + false negatives) and specificity (true negatives/true negatives + false positives), was determined using the Youden index (Sensitivity + Specificity − 1). The 95% confidence intervals were calculated using the Clopper–Pearson exact binomial method. A combination biomarker analysis was performed using a logistic regression model. Statistical analyses were conducted in GraphPad Prism (v8.0; GraphPad Software Inc., San Diego, CA, USA), and results with *p* ≤ 0.05 were deemed statistically significant.

## 3. Results

### 3.1. Characteristics of the Participants

The study included 15 LC patients (NSCLC only) and 30 healthy controls (HC), equally divided into low radon (LR, n = 15) and high radon (HR, n = 15) exposure groups, with a mean age of 61.5 ± 12.3 years. Table 1 summarizes the demographic and clinical characteristics of the study population from Kong Khaek subdistrict, including age, gen-der, education level, alcohol consumption, smoking status, cancer stage, and tumor histology. All participants were either never smokers or former smokers who had resided in the study areas for at least 15 years. Smoking status differed significantly among the groups (*p* = 0.004), with former smokers observed exclusively in the LC group. Among LC patients, most were diagnosed at stage I (86.7%), while the remaining were stage II (13.3%). Histologically, two-thirds of LC patients had adenocarcinoma (66.7%) and one-third had squamous cell carcinoma (33.3%). No statistically significant differences were observed in age, gender, education, or alcohol use across the three groups, and all participants were non-smokers, with former smokers found only in the LC group.

### 3.2. Comparative Serum Analyte Levels in LC Versus HC Groups

**Figure 1:**
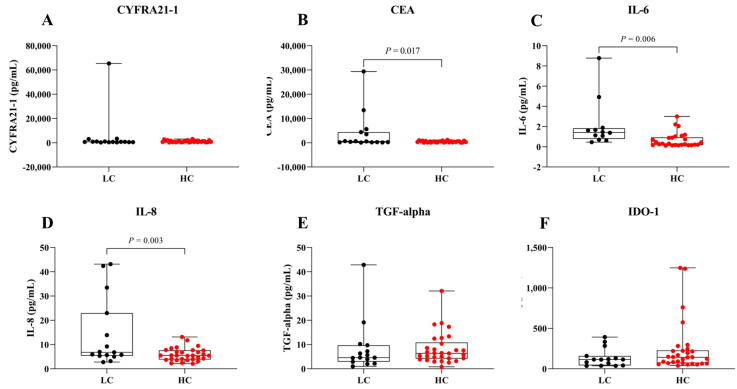
Serum levels in LC and HC groups. Box plots represent the distribution of (**A**) CYFRA 21-1, (**B**) CEA, (**C**) IL-6, (**D**) IL-8, (**E**) TGF-alpha, and (**F**) IDO-1 levels in both groups.

Figure 1 shows serum levels of CYFRA 21-1 (A), CEA (B), IL-6 (C), IL-8 (D), TGF-alpha (E), and IDO-1 (F) in LC and HC groups. Data are presented as box plots displaying the median and range (Figure 1 and Table 2). The minimal detectable concentrations were as follows: CYFRA 21-1: 274.9 pg/mL, CEA: 62.2 pg/mL, IL-6: 0.1 pg/mL, IL-8: 2.1 pg/mL, TGF-alpha: 0.8 pg/mL and IDO-1: 37.2 pg/mL. The intra and inter-assay variabilities were <5 and 15–20%, respectively. Analysis revealed that serum levels of CEA, IL-6, and IL-8 were significantly higher in the LC group compared to the HC group (*p* < 0.05). However, no significant differences were observed for serum CYFRA 21-1, TGF-alpha, or IDO-1 levels between LC and HC groups.

### 3.3. Comparative Levels of Serum Analytes Among LC, LR, and HR Groups

**Figure 2:**
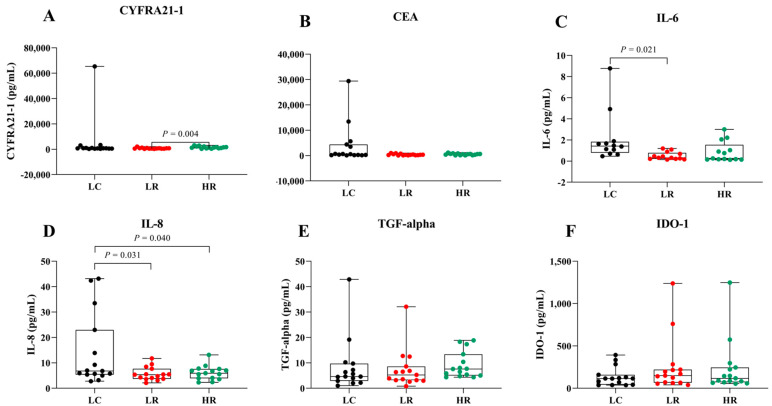
Serum levels of biomarkers in LC, LR, and HR groups. Box plots represent the distribution of (**A**) CYFRA 21-1, (**B**) CEA, (**C**) IL-6, (**D**) IL-8, (**E**) TGF-alpha, and (**F**) IDO-1 across the study groups.

To assess the potential of serum biomarkers for LC detection, serum levels of CYFRA 21-1, CEA, IL-6, IL-8, TGF-alpha, and IDO-1 were compared among the LC, LR, and HR groups (Figure 2 and Table 2). Data are presented as box plots indicating the median and overall range. Serum CYFRA 21-1 levels were significantly different between the LR and HR groups (*p* = 0.004). Serum IL-8 was significantly elevated in LC patients compared to the HR group (*p* = 0.04). Additionally, IL-6 and IL-8 levels tended to be higher in LC patients compared to the other groups, whereas CEA, TGF-alpha, and IDO-1 did not show significant differences across groups. These findings suggest that IL-6 and IL-8 may serve as biomarkers for LC in radon-exposed populations, while CYFRA 21-1 could differentiate between varying radon exposure levels.

### 3.4. Evaluation of the Diagnostic Capability of Serum Biomarkers for LC Risk in HC and HR Groups

**Figure 3:**
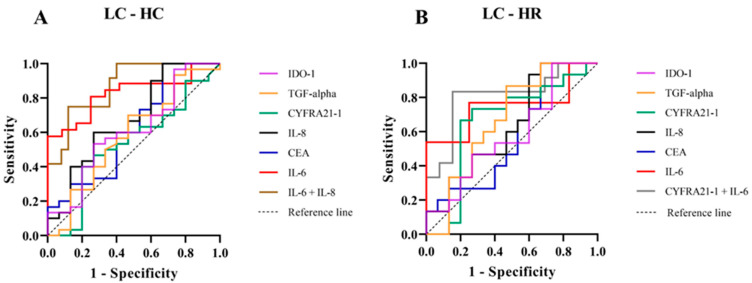
Receiver Operating Characteristic (ROC) curves for serum biomarkers in LC compared with HC groups (**A**) and LC compared with HR groups (**B**). The ROC curves depict the diagnostic performance of IDO-1 (purple), TGF-alpha (orange), CYFRA 21-1 (green), IL-8 (black), CEA (blue), IL-6 (red), the combination of IL-6 and IL-8 (brown), and the combination of CYFRA 21-1 and IL-6 (gray). Sensitivity is plotted against 1-specificity, with the diagonal dashed line representing the reference.

We assessed the potential of serum biomarkers to differentiate LC from the HC group using receiver operating characteristic (ROC) analysis. The key diagnostic parameters, including the area under the ROC curve (AUC), sensitivity, specificity, and Youden index with their 95% confidence intervals (CI), are summarized in Figure 3A and Table 3. Among the individual biomarkers, IL-6 demonstrated the highest discriminative performance (AUC = 0.83, 95% CI: 0.70–0.96), with a sensitivity of 0.62 (95% CI: 0.36–0.80), a specificity of 0.92 (95% CI: 0.79–0.98), and a Youden index of 0.53 (95% CI: 0.14–0.78). IL-8 also exhibited moderate discriminative ability (AUC = 0.68, 95% CI: 0.50–0.85), with a sensitivity of 0.40 (95% CI: 0.20–0.64), a specificity of 0.87 (95% CI: 0.70–0.95), and a Youden index of 0.27 (95% CI: −0.10–0.59). In contrast, CYFRA 21-1, CEA, TGF-alpha, and IDO-1 showed limited diagnostic performance, with AUC values ranging from 0.53 to 0.62. The combination of IL-6 and IL-8 significantly improved the overall diagnostic accuracy, achieving the highest AUC (0.87, 95% CI: 0.76–0.99) and markedly enhancing specificity [0.96 (95% CI: 0.83–0.99)]; however, it did not substantially improve sensitivity [0.41 (95% CI: 0.20–0.64)]. These results suggest IL-6 alone provides strong discriminative ability between LC and HC groups, while the IL-6 and IL-8 combination primarily enhances specificity and overall accuracy without significantly affecting sensitivity.

Furthermore, the HR group, which warranted particular attention due to their elevated risk, was further examined for a potential association with LC. The diagnostic performance of serum biomarkers in distinguishing LC from HR individuals, as assessed by ROC analysis, is presented in Figure 3B and Table 4. Among individual biomarkers, IL-6 showed the strongest discriminative ability (AUC = 0.75, 95% CI: 0.55–0.95), with a sensitivity of 0.54 (95% CI: 0.30–0.75), specificity of 0.92 (95% CI: 0.70–0.99), and a Youden index of 0.46 (95% CI: 0.00–0.74). CYFRA 21-1 ranked second (AUC = 0.66, 95% CI: 0.45–0.87), with a sensitivity of 0.67 (95% CI: 0.42–0.85), specificity of 0.80 (95% CI: 0.55–0.93), and a Youden index of 0.47 (95% CI: −0.03–0.78). Importantly, the combined use of IL-6 and CYFRA 21-1 significantly enhanced overall diagnostic performance, yielding the highest AUC (0.81, 95% CI: 0.63–0.99), sensitivity of 0.75 (95% CI: 0.48–0.89), specificity of 0.85 (95% CI: 0.62–0.96), and Youden index of 0.60 (95% CI: 0.10–0.85). Other biomarkers (CEA, IL-8, TGF-alpha, IDO-1) exhibited moderate to limited diagnostic values (AUC: 0.58–0.67 Youden index: 0.13–0.20). Our findings indicate that IL-6 is the most effective individual biomarker for differentiating LC from HR. The combination of IL-6 and CYFRA 21-1 substantially improves diagnostic accuracy, supporting the use of combined biomarkers to enhance LC detection in high-risk populations chronically exposed to elevated radon levels.

## 4. Discussion

Radon gas is recognized as a significant and well-established environmental risk factor for LC development, especially among non-smokers [12,13]. Most LC cases are diagnosed at advanced stages, often leading to poor prognosis [3,4]. Therefore, early detection is essential, particularly in high-risk populations residing in high radon areas who are non-smokers. Although our previous study demonstrated that serum CYFRA 21-1 and CEA could differentiate LC patients from individuals exposed to high radon levels [15], their diagnostic performance remains suboptimal, warranting further investigation. Currently, no single biomarker reliably provides sufficient sensitivity or specificity for LC screening related to chronic radon exposure [21]. Therefore, combining multiple serum biomarkers may offer improved sensitivity and accuracy in identifying LC risk among individuals chronically exposed to high radon levels.

In this study, serum levels of CEA, IL-6, and IL-8 were significantly higher in the LC group compared to the HC group (*p* < 0.05). In contrast, serum levels of CYFRA 21-1, a traditional clinical LC marker, were also elevated but did not reach statistical significance (Figure 1). The significant elevation of IL-6 and IL-8 highlight their potential roles as biomarkers for early LC detection (Figure 1C,D). These pro-inflammatory cytokines may reflect tumor-promoting inflammation and microenvironmental changes occurring in the early stages of lung carcinogenesis [7,12,21,22,23,24]. Increased CEA levels in the LC group (Figure 1B) likely reflect enhanced secretion by cancer cells and tumor-associated processes [25,26]. Nevertheless, the lack of significant results for CYFRA 21-1 between the LC and HC groups (Figure 1A) could reflect the combined effects of tumor heterogeneity, variability in biomarker expression across disease stages, and sample size limitations [27,28]. In addition, no significant difference in serum TGF-alpha levels was observed between the LC and HC groups (Figure 1E), which may be attributed to the fact that TGF-alpha is frequently expressed in both normal and tumor tissues, and due to its involvement in autocrine signaling, its levels may not significantly increase in the blood circulation [29,30]. Interestingly, the serum levels of IDO-1 were lower in the LC group compared to HC group (Figure 1D). This finding contrasts with previous studies reporting elevated IDO-1 expression in tumor tissues, where it contributes to immune suppression and tumor progression through tryptophan catabolism and kynurenine production [31,32]. The inverse trend observed in our serum data may suggest that IDO-1 activity is largely confined to the tumor microenvironment, limiting its systemic release, or that compensatory immune mechanisms reduce circulating IDO-1 levels in LC patients. Alternatively, decreased serum IDO-1 could reflect systemic immune alterations such as T-cell dysfunction or changes in dendritic cell activity [33,34]. A deeper exploration of these immunological mechanisms, including the balance between local versus systemic IDO-1 activity, would provide valuable insights and should be addressed in future studies. Therefore, serum TGF-alpha and IDO-1 levels may lack sufficient sensitivity to serve as reliable biomarkers for distinguishing LC patients from healthy individuals. Taken together, these findings indicate that serum IL-6, IL-8, CEA and CYFRA21-1 may serve as potential biomarkers for distinguishing LC from HC groups. The diagnostic potential of these biomarkers was further evaluated using ROC analysis (Figure 3A and Table 3). Among individual biomarkers, IL-6 exhibited the highest discriminative performance [AUC = 0.83 (95% CI: 0.70–0.96), sensitivity = 0.62 (95% CI: 0.36–0.80), specificity = 0.92 (95% CI: 0.79–0.98), Youden index = 0.53 (95% CI: 0.14–0.78)], followed by IL-8 [AUC = 0.68 (95% CI: 0.50–0.85), sensitivity = 0.40 (95% CI: 0.20–0.64), specificity = 0.87 (95% CI: 0.70–0.95), Youden index = 0.27 (95% CI: −0.10–0.59)]. These results align with previous findings reported in the literature [35,36]. Although combining IL-6 and IL-8 improved the overall specificity and AUC, it did not significantly enhance sensitivity or the Youden index [AUC = 0.87 (95% CI: 0.76–0.99), sensitivity = 0.41 (95% CI: 0.20–0.64), specificity = 0.96 (95% CI: 0.83–0.99), Youden index = 0.37 (95% CI: 0.03–0.64)]; however, it still provided a statistically significant distinction between the LC and HC group (*p* = 0.0003). This outcome may be explained by the fact that IL-6 and IL-8 both being pro-inflammatory cytokines with overlapping roles in LC progression, thus limiting their independent predictive value [7,22,23,24].

Additionally, we focused on the HR group, owing to their heightened risk of LC, to evaluate the diagnostic value of serum biomarkers in distinguishing them from patients with LC. Serum IL-8 levels were significantly higher (*p* = 0.04) in the LC group compared to the HR group, whereas CYFRA 21-1, CEA, and IL-6 showed elevated but statistically non-significant differences (Figure 2 and Table 2). No significant differences were observed in serum TGF-alpha and IDO-1 levels across groups (Figure 2E,F). Remarkably, serum CYFRA 21-1 was the only biomarker significantly different between the HR and LR groups (Figure 2A). The higher serum CYFRA 21-1 levels observed in the HR group compared to the LR group (*p* = 0.004) may reflect prolonged radon exposure, suggesting its potential as a biomarker for radiation-induced lung injury. This association is likely mediated by radon-emitted alpha particles that cause DNA damage and oxidative stress, leading to lung epithelial injury. Subsequent tumor cell breakdown releases cytokeratin 19 fragments, thereby elevating serum CYFRA 21-1 levels [6,8,12]. These findings align with previous studies indicating elevated CYFRA 21-1 levels as a biomarker for LC development in never-smokers exposed to high radon levels [15,37].

To validate the diagnostic performance of potential serum biomarkers for assessing LC risk in the HR group, we performed ROC analysis. Among individual biomarkers, IL-6 exhibited the highest AUC [0.75 (95% CI: 0.55–0.95)], while CYFRA 21-1 showed the highest Youden index [0.47 (95% CI: −0.03–0.78)] (Figure 3B and Table 4). Serum IL-6 is a pro-inflammatory cytokine involved in immune responses and tumor progression [22,23,24]. Chronic radon exposure has been associated with elevated IL-6 expression, which may contribute to LC development and metastasis [38]. In this context, radon decay releases alpha particles that induce DNA damage and oxidative stress in lung epithelial cells and immune cells. This cellular damage stimulates the release of pro-inflammatory cytokines, including IL-6, whose elevated levels can promote cell proliferation, angiogenesis, and the survival of damaged cells, processes closely linked to lung carcinogenesis [12,22,23,24,38,39]. Importantly, the combined use of serum IL-6 and CYFRA 21-1 significantly enhanced diagnostic performance (*p* = 0.01), yielding an increased AUC of 0.81 (95% CI: 0.63–0.99), sensitivity of 0.75 (95% CI: 0.48–0.89), specificity of 0.85 (95% CI: 0.62–0.96), and a Youden index of 0.60 (95% CI: 0.10–0.85). Moreover, when evaluating the combined use of serum IL-6 and CYFRA 21-1 between the LC and HR groups, and serum IL-6 and IL-8 between the LC and HC groups (Figure 3A,B), the diagnostic performance of these biomarkers for LC risk becomes apparent. Notably, serum CYFRA 21-1 emerges as a key discriminator between the LR and HR groups relative to the HC group, which is consistent with the experimental results shown in Figure 2A. These findings highlight the crucial roles of IL-6 and CYFRA 21-1 in mediating radon-induced lung carcinogenesis and emphasize their potential utility as biomarkers for LC risk assessment in individuals residing in high-radon areas. Further investigations are warranted to elucidate the underlying mechanisms of radon-related inflammation and lung carcinogenesis, with particular focus on DNA damage, oxidative stress, and the pathways associated with IL-6 and CYFRA 21-1.

A key strength of this study is its focus on a non-smoking population residing in high-radon areas for at least 15 years, with indoor radon concentrations systematically measured over a six-month period. This design allows direct investigation of health effects resulting from long-term radon exposure. Another notable strength is that the participants were selected from our previous metabolomics studies [18,19], where significant metabolite differences were identified among the LC, HR, and LR groups. This consistency enhances the reliability of our findings and reinforces the link between metabolic alterations and LC risk in populations chronically exposed to radon. Consistent with previous reports, alterations in sphingolipid metabolism, particularly involving sphingosine and its derivatives, have been associated with LC [40]. The interplay between sphingosine and IL-6 may contribute to tumor progression and cancer-related inflammation [41]; however, the direct relationship between sphingosine metabolites and CYFRA 21-1 remains unclear. Further research is warranted to clarify these associations. Nevertheless, this study has several limitations. First, the relatively small sample size may restrict the generalizability of our findings, necessitating larger-scale studies for validation and deeper exploration. Second, the elevated PM levels in the study area might interact with radon exposure, potentially confounding specific radon-induced health effects. Third, the absence of detailed analyses by NSCLC subtype and tumor stage limits the interpretation of biomarker variability across these clinical parameters. Fourth, multiple comparisons were performed without formal adjustment, and the results should therefore be interpreted with caution. Fifth, cut-off values for the IL-6 + IL-8 and IL-6 + CYFRA 21-1 combinations could not be determined due to software limitations, and the exploratory logistic regression models were retained without cross-validation while the analysts were not blinded to group allocation; both are acknowledged as methodological constraints. Finally, because radon exposure from natural background radiation is ubiquitous and unavoidable, a true unexposed control group cannot be identified; thus, controlled cell or animal model studies may be required.

## 5. Conclusions

To the best of our knowledge, this is the first study to examine the combined use of IL-6 and CYFRA 21-1 as potential biomarkers for lung cancer screening among individuals with high residential radon exposure in Thailand. The results suggest that these biomarkers, especially when used in combination, may improve diagnostic accuracy in this high-risk population. However, additional research, including controlled cell and animal studies, is warranted to confirm their clinical applicability and effectiveness.

## Figures and Tables

**Table 1:** Baseline characteristics of study participants.

Characteristics	LC (n = 15)	LR (n = 15)	HR (n = 15)	*p* Value
Age in years, Mean (SD)	62 (13.3)	61.1 (11.2)	61.3 (13)	0.98
Gender				1
Male	8	8	8	
Female	7	7	7	
Education				0.91
Middle school or lower	9	10	9	
High school or higher	6	5	6	
Alcohol consumption				0.84
Yes	2	3	2	
No	13	12	13	
Smoking status				0.004
Never smokers	10	15	15	
Former smokers	5	0	0	
Stage of lung cancer				-
I	13	0	0	
II	2	0	0	
Tumor histology				-
Adenocarcinoma	10	0	0	
Squamous cell carcinoma	5	0	0	

**Table 2:** Comparative levels of serum analytes among LC and HC groups (LR and HR).

Markers (pg/mL)	LC (n = 15)	HC (n = 30)	LR (n = 15)	HR (n = 15)
CYFRA 21-1				
Mean	5325.8	1132.5	752	1513.1
SD	16,631.1	766.2	470.5	827.3
Min	274.9	274.9	274.9	346.4
Max	65,353.5	3151.4	1321	3151.4
Median	778.1	876.4	609	1392.7
CEA				
Mean	3990	459.8	391.7	527.9
SD	7882.8	312.6	305.6	314.8
Min	121.2	62.2	62.2	107.6
Max	29,384.8	1174.9	1010.2	1174.9
Median	568.8	377.7	316.6	572.6
IL-6				
Mean	2.2	0.7	0.5	0.9
SD	2.4	0.7	0.4	1
Min	0.5	0.1	0.1	0.1
Max	8.8	3	1.2	3
Median	1.4	0.3	0.3	0.3
IL-8				
Mean	14.2	5.9	5.7	6.1
SD	14.2	2.7	2.6	2.8
Min	2.7	2.1	2.1	2.3
Max	43.1	13.1	11.8	13.1
Median	6.8	5.5	5.4	5.9
TGF-alpha				
Mean	8.5	8.3	7.4	9.2
SD	10.5	6.5	7.7	5.2
Min	1	0.8	0.8	4.4
Max	42.9	32.1	32.1	18.9
Median	4.7	6.4	5.3	7.6
IDO-1				
Mean	138.3	243.4	252.9	233.8
SD	111.8	312.6	324.1	311.7
Min	37.2	39.9	39.9	54.8
Max	393.2	1248.1	1238.2	1248.1
Median	115.4	143.4	149.8	118.4

**Table 3:** Comparison of the diagnostic performance of serum biomarkers in LC and HC groups, including AUC, sensitivity, specificity, Youden index, and their 95% confidence intervals (CI).

Biomarkers	Cut-Off	AUC (95% CI)	Sensitivity (95% CI)	Specificity (95% CI)	Youden Index (95% CI)	*p* Value
CYFRA 21-1	1157.00	0.53 (0.35–0.72)	0.40 (0.20–0.64)	0.80 (0.63–0.90)	0.20 (−0.17–0.55)	0.72
CEA	149.20	0.62 (0.43–0.80)	0.20 (0.07–0.45)	0.93 (0.79–0.98)	0.13 (−0.14–0.43)	0.20
IL-6	0.55	0.83 (0.70–0.96)	0.62 (0.36–0.80)	0.92 (0.79–0.98	0.53 (0.14–0.78)	0.001
IL-8	4.90	0.68 (0.50–0.85)	0.40 (0.20–0.64)	0.87 (0.70–0.95)	0.27 (−0.10–0.59)	0.06
TGF-alpha	10.31	0.58 (0.40–0.77)	0.27 (0.11–0.52)	0.87 (0.70–0.95)	0.13 (−0.19–0.47)	0.37
IDO-1	136.40	0.61 (0.43–0.79)	0.53 (0.30–0.75)	0.73 (0.56–0.86)	0.27 (−0.14–0.61)	0.24
IL-6 + IL-8		0.87 (0.76–0.99)	0.41 (0.20–0.64)	0.96 (0.83–0.99)	0.37 (0.03–0.64)	0.0003

**Table 4:** Comparison of the diagnostic performance of serum biomarkers in LC and HR groups, including AUC, sensitivity, specificity, Youden index, and their 95% confidence intervals (CI).

Biomarkers	Cut-Off	AUC (95% CI)	Sensitivity (95% CI)	Specificity (95% CI)	Youden Index (95% CI)	*p* Value
CYFRA 21-1	1157.00	0.66 (0.45–0.87)	0.67 (0.42–0.85)	0.80 (0.55–0.93)	0.47 (−0.03–0.78)	0.13
CEA	149.20	0.58 (0.37–0.79)	0.20 (0.07–0.45)	0.93 (0.70–0.99)	0.13 (−0.23–0.44)	0.47
IL-6	0.54	0.75 (0.55–0.95)	0.54 (0.30–0.75)	0.92 (0.70–0.99)	0.46 (0.00–0.74)	0.03
IL-8	4.54	0.64 (0.43–0.84)	0.33 (0.15–0.58)	0.87 (0.62–0.96)	0.20 (−0.23–0.55)	0.21
TGF-alpha	10.31	0.67 (0.47–0.87)	0.33 (0.15–0.58)	0.87 (0.62–0.96)	0.20 (−0.23–0.55)	0.11
IDO-1	137.40	0.59 (0.38–0.79)	0.47 (0.25–0.70)	0.73 (0.48–0.89)	0.20 (−0.27–0.59)	0.42
CYFRA21-1 + IL-6		0.81 (0.63–0.99)	0.75 (0.48–0.89)	0.85 (0.62–0.96)	0.60 (0.10–0.85)	0.01

## Data Availability

The data presented in this study are available from the authors on reasonable request.

## Abbreviations

The following abbreviations are used in this manuscript:

AUC	Area under the curve
CEA	Carcinoembryonic antigen
CI	Confidence interval
CYFRA 21-1	Cytokeratin 19 fragment
HC	Healthy controls
HR	High radon
IDO-1	Indoleamine 2,3-dioxygenase 1
IL-6	Interleukin-6
IL-8	Interleukin-8
LC	Lung cancer
LDCT	Low-dose computed tomography
LOD	Limit of detection
LR	Low radon
NSCLC	Non-small cell lung cancer
PM	Particulate matter
ROC	Receiver operating characteristic
SCLC	Small cell lung cancer
SD	Standard deviation
TGF-alpha	Transforming growth factor alpha
